# Abortion experience and self-efficacy: exploring socioeconomic profiles of GHANAIAN women

**DOI:** 10.1186/s12978-019-0775-9

**Published:** 2019-07-26

**Authors:** Nkechi Srodah Owoo, Monica P. Lambon-Quayefio, Nicole Onuoha

**Affiliations:** 1 Department of Economics, University of Ghana, P. O. Box LG 57, Legon, Accra Ghana; 2 Boston, USA

**Keywords:** Abortion, Self-efficacy, Spatial hot spots, GIS, Ghana maternal health survey

## Abstract

**Background:**

Unsafe abortions remain a major global public health concern and despite its prevalence, unsafe abortions remain one of the most neglected global health challenges. The proportion of women in Ghana who have experienced unsafe abortions has increased from 45% in 2007 to 62% in 2017. Given the noted consequences of (unsafe) abortions on women health, it is important to explore factors correlated with women’s abortion decisions and why they opt for safe or unsafe methods. The study also examines determinants of over 6,000 Ghanaian women’s self-efficacy in abortion decision-making, given that this is likely to affect the likelihood of future abortions.

**Methods:**

Using cluster-level Geographic Information System data from the 2017 Ghana Maternal Health Survey, the study provides a hot spot analysis of the incidence of abortion in the country. The study also makes use of Probit multivariate analyses also show the correlates of abortion with socio-economic factors.

**Results:**

Results suggest that abortion among women is positively correlated with the absence of partners, low education levels, higher household wealth, lower parity and family size, polygyny and Christian religious background.

**Conclusion:**

It is observed that the groups of women with higher abortion self-efficacy are the same groups of women who are more likely to opt for safer abortion methods, indicating some correlation, albeit indirect, between abortion self-efficacy and women’s abortion behaviors in Ghana. Relevant policy applications are adduced from these research findings.

## Plain English summary

Unsafe abortions remain a major global public health concern and despite its prevalence, unsafe abortions remain one of the most neglected health challenges. The proportion of women in Ghana who have experienced unsafe abortions has increased significantly between 2007 and 2017. In view of the consequences of (unsafe) abortions on women health, it is important to understand the factors that influence women’s abortion decisions and the reasons why women opt for unsafe methods. The study also examines the decision-making power of Ghanaian women in relation to abortion decisions as this may have implications for the likelihood of future abortion decisions. Using information from the most recent Maternal Health Survey in Ghana, the study provides a geographic analysis of the incidence of abortion in the country. Results from the analysis show that abortion among women is positively associated with the absence of partners, low education levels, higher household wealth, lower parity and family size, polygyny and Christian religious background. It is observed that the groups of women with higher abortion decision-making power are the same groups of women who are more likely to opt for safer abortion methods. This is indicative of a relationship between abortion decision-making power and women’s abortion behaviours in Ghana. These research findings provide relevant policy applications.

## Background

According to World Health Organisation, WHO [51], an unsafe abortion is the termination of a pregnancy by people lacking the necessary skills, or in an environment lacking minimal medical standards, or both. Unsafe abortions remain a major global public health concern and despite its prevalence, unsafe abortions remain one of the most neglected global health challenges. Between 2010 and 2014, 25.1 million women experienced an ‘unsafe’ abortion. Of these, an estimated 8.2 million abortions occurred in Africa [24]. The rates of abortion vary between sub-regions in the continent: 31 per 1,000 women in West Africa; 34 per 1,000 women in East, Middle and Southern Africa; and 38 per 1,000 women in Northern Africa, with slightly higher rates for unmarried women [47]. An estimated 19–20 million unsafe abortions are carried out annually [27], and nearly all of these (approximately 98%) take place in developing countries [45]. About 68,000 women die each year as a result of hemorrhage, infection and poisoning from these unsafe abortions [27]. Many millions other women experience morbidity outcomes. Although these statistics are staggering, it is noteworthy to emphasize that they may be understated as a result of the under-reporting of abortion by women, likely in efforts to avoid stigmatization [28].

Abortion, particularly in cases where they are unsafe, may have serious health consequences ([18, 36]) and, in the most severe circumstances, may lead to death [3, 42]. In developing countries, unsafe abortions are noted to be the leading cause of maternal mortality, where over 99% of all global abortion-related deaths occur [27]. In Ghana, one of the largest contributors of maternal mortality is complications resulting from unsafe abortions [26]. The current maternal mortality rate in the country is 319 per 100,000 women [32] and deaths from abortions constitute approximately 15–30% of maternal mortality [5]. According to the 2007 and 2017 Ghana Maternal Health Surveys (GMHS) conducted in the country, the percentage of abortions performed by untrained or unauthorized providers has increased from 40 to 57% within the period. The proportion of women who had undergone unsafe abortions was 45% in 2007 [49] but increased to 62% by 2017, according to statistics from the recent wave of the GMHS. This has implications for women’s morbidity and mortality outcomes.

Although abortion is not legal in Ghana, the laws may be perceived to be relatively liberal, compared to the case in a number of other African countries. In Ghana, abortion may be performed in cases where the pregnancy is the result of rape or incest; in an attempt to protect the mental or physical health of the mother; or in the event of a malformation of the fetus. From a policy perspective, Ghana did not incorporate safe abortion into its reproductive health policy until 2003 [22]. In 2006, the Ghana Health Service introduced the new standards and protocols for safe abortion services which included some room for the interpretation of Ghana’s abortion law. To a large extent however, the law continues to be interpreted as prohibitive with implications for access to abortion services. Although the national health insurance scheme (NHIS) does not cover abortions, it does cover health care services for complications arising out of abortion operations. Post-abortion care is however not covered under NHIS. It is a common notion among citizens in Ghana that the national social insurance scheme needs to do more to prevent negative outcomes of unsafe abortions in the country.

The third Sustainable Development Goal aims to reduce maternal mortality by 2030. In Ghana, it is unlikely that this goal will be achieved without adequate focus on the issue of abortion. It is important to understand the factors that contribute to women’s abortion-seeking behaviors. Additionally, studies should interrogate factors that affect the choice of a safe or unsafe abortion method. Self-efficacy is rapidly becoming recognized as an important determinant of women’s reproductive health decisions. Self-efficacy may be described as the belief we have in our own abilities, specifically our ability to meet the challenges ahead of us and complete a task successfully. Although most commonly applied to studies on contraceptive use, it is equally imperative to understand the factors that affect women’s abortion self-efficacy. This is because belief in one’s ability tends to have a profound effect on these abilities [7]. Women who feel that they are able to take sole decisions on their own reproductive health may be more likely to accomplish these goals. Self-efficacy may therefore be relevant for predicting women’s future abortion-seeking behaviors.

The three questions examined in the present research are as follows:

- What factors explain women’s experience of abortion in Ghana?
- What are the determinants of safe abortion practices among women?
- What variables are related with women’s abortion self-efficacy?
  - Is there a relationship, however indirect, between abortion self-efficacy and women’s choice of safe/unsafe abortion methods?

The present study makes a significant contribution to the existing literature on women’s abortion behaviors in Ghana, and other countries, in a number of ways. First, with the exception of the paper by Sundaram et al. [49], a number of other studies on abortion in Ghana have tended to be hospital-based and in such cases, lack representativeness [2]. In few cases where population-based surveys are employed, these have typically focused on small geographical areas [1]. The present study uses nationally-representative data to explore women’s social, economic, individual and institutional determinants of the decision to seek an abortion and how safely this is carried out. Second, the study provides a spatial mapping of the incidence of abortion in the country, which makes it easier for policy targeting and interventions. Third, the study investigates the factors relating to women’s perception of their abilities to take abortion-related decisions. This is important because higher abortion efficacy among women may increase the likelihood of future abortions and women’s choice of safe or unsafe abortion methods. Self-efficacy has been studied more frequently in relation to women’s contraceptive use decision-making processes, likely as a result of limited data on abortion. Indeed, self-efficacy has been shown to play an important role in women’s use of and adherence to family planning methods [34, 50]. No studies, to the best of knowledge, have examined abortion self-efficacy among women despite potential effects on abortion decision-making processes.

### Brief literature on Abortion decision-making processes

According to Coast et al. [13], women’s abortion decision-making may be understood within three main contexts: abortion-specific experiences, individual domains and national/ institutional contexts. *Abortion-specific experiences* may include women’s access to health facilities for pregnancy testing and relevant diagnosis [48]; in addition to the ability to disclose the information to partner, family, friends, etc. with attendant implications. For instance, people to whom women disclose pregnancy information may influence their abortion decisions [13, 44]. The ability to access resources for abortion may also influence women’s abortion decisions. Economic resources have been identified as a critical determinant [31, 52]. Women who feel financially unprepared, as a result of low wealth status of households, may opt for abortions [25]. Access to financial resources may also play a critical role in whether or not a woman opts for a safe abortion method [33]. Women’s perceived and experienced outcomes from abortions may also affect their decision-making process. Child spacing may also be an important consideration and women who feel that the timing of births is inadequate may opt for an abortion [37].

The woman’s *individual domain* also influences whether and how she chooses to seek abortion-related care. Socioeconomic, demographic and health characteristics such as age [12], household wealth [49], education [17], ethnicity [16], among others are likely to play a critical role in abortion decision-making processes. Additionally, beliefs about the godliness of abortion [14, 15, 30, 43] may be important. In their research on China, Liang et al. [30] found that among Muslims, a baby is considered to be a gift from Allah and therefore terminating a pregnancy is a sin. In this regard, a stillborn baby or even a fetus from a miscarriage is revered with a naming ceremony and a burial, a testament to the high value assigned to an unborn child. In Ghana, some medical practitioners refuse to perform abortions because these are considered as murder and therefore a sin against God. Additionally, here also, pregnancies are considered to be a gift from God and should therefore be accepted, even if they are unintended [4]. These belief systems and synonymy of abortions with murder and sin are likely to discourage abortion-seeking care among many women.

The *national or institutional context* also matters for decisions relating to abortion care. The legal environment may pose an important constraint to abortion-seeking decisions. In many countries, laws on abortion may be heterogenous and vary based on factors such as abortion methods used, gestational age, among others [9]. Inaccurate knowledge of the laws surrounding abortion may therefore lead to otherwise willing providers failing to provide this service [40] or clandestine delivery of services despite legal restrictions [38]. The health system is another important determinant of abortion-seeking behavior. Financing may play a critical role and affect both the decision to have an abortion, in addition to the safety of the process [23]. Technology such as the mass media that influences knowledge about abortion methods, side effects and promotes general discourse about abortion stigma, among other issues, is another factor expected to affect women’s decisions relating to abortion [39]. The socio-cultural context comprising family size and birth spacing may also be expected to be important [10].

### Literature on (Abortion) self-efficacy

According to Bandura [7], self-efficacy may be described as the belief we have in our own abilities, specifically our ability to meet the challenges ahead of us and complete a task successfully. The theory of self-efficacy is largely rooted in the psychology literature and is discussed expansively in Bandura’s [6] seminal paper. According to Bandura’s [7] social cognitive theory, self-efficacy may be enhanced through a number of channels. First, through repeated experiences- women who may have undergone abortion experiences in the past may be more confident in their abilities to seek other procedures in the future, especially if there were few negative outcomes from earlier experiences. Second, self-efficacy may be affected by the experiences of others. A woman who observes similar women undergo an abortion and emerge successfully, may have a stronger belief in her own ability to also undergo the procedure.

Social persuasion is another channel through which self-efficacy may be developed. When women are persuaded (e.g. higher education) that they possess the necessary abilities to succeed, they are more likely to develop stronger beliefs in their own capabilities to handle a particular situation. Finally, emotional and psychological proclivities may also affect one’s self-efficacy. These may be related to the presence of partner support, religious and cultural surroundings, among others. Therefore, sources of self-efficacy may include familial, peer-influences and own-experiences, and knowledge of this is useful to understanding women’s abortion behaviors in Ghana.

Self-efficacy has been found to affect women’s reproductive health behaviors in a number of ways. A number of researchers have found that women’s use of contraceptives is directly related to their degree of self-efficacy. For instance, using data on the United States, Tomaszewski et al. [50] found that women with higher self-efficacy were more likely to patronize and adhere to their contraceptive regimes. Muhindo et al. [34] also find that among Ugandan women, low self-efficacy is a predictor of poor contraceptive adherence. Although no studies, to the best of knowledge, have examined the determinants of abortion self-efficacy among women, it is reasonable to expect that this would be directly related to both present and future abortion practices. It is therefore a subject-matter worth exploring.

## Methods

The study employs the second wave of the 2017 Ghana Maternal Health Survey for the analyses of these relationships. The data collection is done by the Ghana Statistical Service, in conjunction with Macro International. The datum is cross-sectional and nationally representative. The 2017 wave includes information on 27,000 households and 25,063 successfully surveyed women between the ages of 15 and 49 years. The Ghana Maternal Health Survey is expected to be complementary to the various waves of the Ghana Demographic and Health Surveys collected.

Birth histories collected from women also provide critical information on over 70,000 births that these women have had. Of the women surveyed, 17,142 women reported that they had ever been pregnant in their lifetime; of these women, 3,702 women had ever an abortion. In order to examine the characteristics of women who had an abortion in the 5 years preceding the survey, the analysis is restricted to the 13,419 women who had a pregnancy within the time frame. The total number of women who had abortions within this 5-year period (i.e. since 2012) is 1,425. A rationale for the restriction of the analysis of women’s abortion experiences to the 5-year period preceding the survey, is to ensure as much alignment as possible between the dependent variable at the time of the survey and other explanatory variables which are likely to vary over time.

The three main dependent variables are binary in nature and include the following: whether a woman had an abortion in the last 5 years preceding the survey; whether she employed a safe abortion procedure; whether a woman feels that she can take independent decisions on abortion i.e. measure of her abortion self-efficacy.

The safety of the abortion process in the present study was measured by both the type of provider and the method of abortion. This construction is consistent with WHO recommendations and guidelines [24]. By law, in Ghana, doctors and nurses/midwives are the only health professionals mandated to extend abortion services to women and therefore women who obtained abortions through these channels were coded as having had ‘safe’ abortions. With respect to methods used, women were coded as having a safe abortion if they employed the following actions to end their pregnancies: dilation and curettage (D&C), manual vacuum aspiration (MVA), and taking Cytotec, mifepristone and misoprostol (medabon, etc.) pills. Other methods like drinking alcohol, herbal concoctions and other home remedies were coded as being unsafe. Taking tablets of an unknown kind were also coded as being unsafe, in addition to injecting the abdomen.

Abortion self-efficacy was present if a woman responded in the affirmative to the question: “Could you decide on your own to get an abortion?” About a quarter of women were noted to have self-efficacy in abortion decision-making. In addition to examining the determinants of abortion self-efficacy, the study also aimed to assess the effect of this self-efficacy on women’s abortion-seeking behaviors. An indirect approach will be taken to achieve this objective however, given that questions on abortion efficacy were posed to only women who had *not* had an abortion. This therefore limits the opportunity and ability to examine more directly, the effects of self-efficacy on present abortion experiences of women. Characteristics of women who seek safe abortion are therefore compared with characteristics of women who have higher self-efficacy in order to establish some correlation, where present.

### Analyses

The empirical strategy consists of both descriptive (graphical and tabular) and econometric analyses of the data. The econometric analyses include multivariate regression models to examine the determinants of women’s likelihood of obtaining an abortion in the last 5 years preceding the survey; the likelihood that a woman has a ‘safe’ abortion; and the factors associated with women’s self-efficacy. Given the binary nature of the dependent variables, a probit model is used and marginal effects are reported.

## Results and discussion

This section comprises four sub-sections- the first section presents a visual representation of the distribution of abortion across Ghana, in addition to a hot/cold spot analysis. The second section provides background characteristics of the study variables used in the analyses while the third section discusses bivariate relationships between unsafe abortions in Ghana and selected women’s socioeconomic characteristics. The final section presents results from multivariate regressions of abortion and abortion self-efficacy on a host of socioeconomic, demographic and geographical variables.

### Spatial analyses

**Fig. 1:**
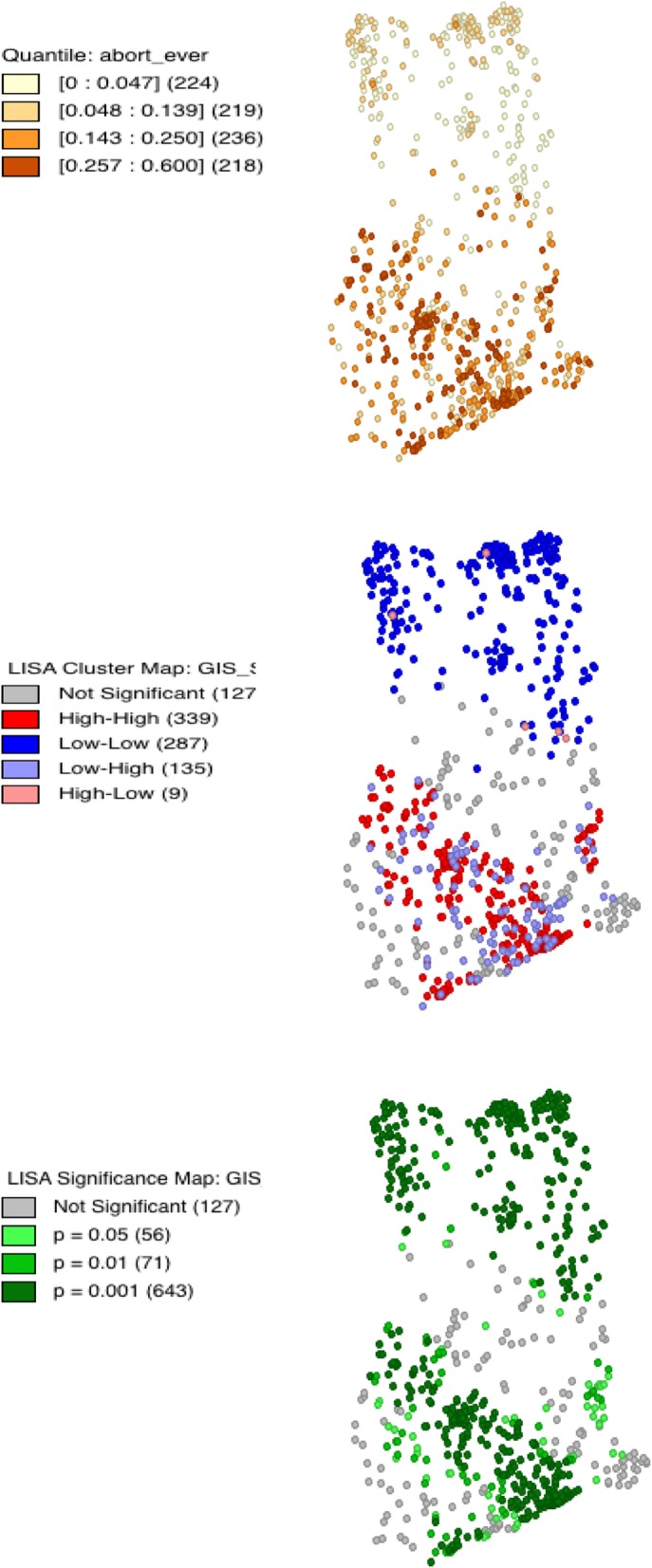
Maps of Ghana Showing Distribution of Abortion, in addition to Significance of Hot and Cold Spots, GMHS, 2017

Figure 1 provides a description of the spatial distribution of abortion in Ghana. Using cluster-level Geographic Information System (GIS) information provided in the 2017 GMHS, the figure shows areas of the country where abortion is most and least prevalent, using hot/cold spot analyses. In the first map, there is a distinct north-south difference in the prevalence of abortion in Ghana- while high incidences of abortion are observed in the southern portion of the country, very low rates are observed in northern Ghana. Areas in the Greater Accra and Ashanti region appear to be hot spots (shown by red dots in the second map) for abortion occurrences. Cold spots (illustrated by blue dots in the second map), with low incidences of abortion, are observed in the northern portions of the country, in regions such as the Northern, Upper East and Upper West regions. The third map shows the significance levels of noted hot and cold spot, with darker shades of green depicting more significant clusters. The hot spot analyses indicate some significant spatial correlation in the incidence of abortion in Ghana.

### Background characteristics

Background characteristics of women is presented in Table 1. From Table 1, although 20% of women report that they have ever had an abortion in their lifetime, only 10% of these occurred in the 5 years preceding the survey. Of the women in the sample who had an abortion, only 37.8% had a safe abortion (i.e. safe method and safe provider).

**Table 1:** Summary Statistics (*weights included*), Ghana Maternal Health Survey, 2017

	Mean	SD	Minimum	Maximum
*Dependent Variables*				
Ever had an abortion	0.196	0.4	0	1
Had an abortion in previous 5 years	0.104	0.3	0	1
Safe abortion (Yes = 1)	0.378	0.49	0	1
Abortion self-efficacy (Yes = 1)	0.255	0.44	0	1
*Explanatory Variables*				
Woman age	29.527	9.7	15	49
Male partner present in household	0.75	0.43	0	1
Household size	5.238	2.85	1	34
*Woman Education levels*				
None/Primary education	0.192	0.39	0	1
Junior secondary school	0.492	0.5	0	1
Senior secondary school	0.222	0.42	0	1
Post-secondary education	0.093	0.29	0	1
No. of children ever born	2.239	2.3	0	14
Household is polygynous (Yes = 1)	0.142	0.35	0	1
*Religion*				
Catholic	0.096	0.29	0	1
Muslim	0.154	0.36	0	1
Other Christian	0.708	0.45	0	1
Traditionalist/ No religion	0.042	0.2	0	1
*Wealth Quintiles*				
Poorest	0.162	0.37	0	1
Poorer	0.188	0.39	0	1
Middle	0.204	0.4	0	1
Richer	0.217	0.41	0	1
Richest	0.228	0.42	0	>1
Woman owns bank account (Yes = 1)	0.295	0.46	0	1
*Mass media*				
Household owns a radio (Yes = 1)	0.766	0.42	0	1
Household owns a TV (Yes = 1)	0.784	0.41	0	1
Urban residence	0.549	0.5	0	1
Access to health facilities not a problem (Yes = 1)	0.763	0.42	0	1
Perception of abortion (Abortion is legal = 1)	0.114	0.32	0	1
Covered by NHIS (Yes = 1)	0.583	0.49	0	1
*Ethnic Groups*				
Akan	0.497	0.5	0	1
Ga	0.083	0.28	0	1
Ewe	0.142	0.35	0	1
Mole Dagbani	0.155	0.36	0	1
Other northern ethnic group	0.124	0.33	0	1
*Region*				
Western	0.129	0.34	0	1
Central	0.089	0.28	0	1
Greater Accra	0.186	0.39	0	1
Volta	0.08	0.27	0	1
Eastern	0.1	0.3	0	1
Ashanti	0.191	0.39	0	1
Brong Ahafo	0.094	0.29	0	1
Northern	0.071	0.26	0	1
Upper East	0.034	0.18	0	1
Upper West	0.025	0.16	0	1
Observations	25062			

Using information largely drawn from the existing literature (described in the preceding section), explanatory variables that are likely to be associated with the main dependent variables are included in the analyses. The average age of women in the sample is 29.5 years. In 75% of cases, a woman’s partner is also resident in the household. The average household size is about 5 members, although a household in the sample has as many as 34 members. Information was collected on the highest education levels that women have attained. 19% of women in the sample either have none or only primary education. About half the sample has a junior secondary school education. Only about 10% of women possess post-secondary education.

Although a woman in the sample has had as many as 14 children, the average number of births or children ever born is 2.2 children. Fourteen percent of women report that their husbands have other wives. With respect to religion, over 70% of the sample is Christian. Information is provided on household wealth status; this is calculated from household ownership of assets and the adoption of a principal component analysis (PCA) technique. About a third of women possess bank accounts.

With respect to mass media, 77 and 78% of women possess radios and televisions, respectively. Fifty-five percent of women reside in urban areas and of the women in the sample, 76% report that distance to health facilities is not a limitation to seeking health care. Although abortion is illegal in Ghana (with the exception of certain special circumstances mentioned above), about 11% of women in the sample perceive it to be legal. Fifty-eight percent of women report that they are covered (not just registered) by the national health insurance scheme (NHIS). With respect to ethnicity, the Akan ethnic group is the most dominant, followed by women who belong to the Mole-Dagbani and other northern ethnic groups. The largest proportion of women surveyed are from the Ashanti and Greater Accra regions.

### Bivariate analyses

**Fig. 2:**
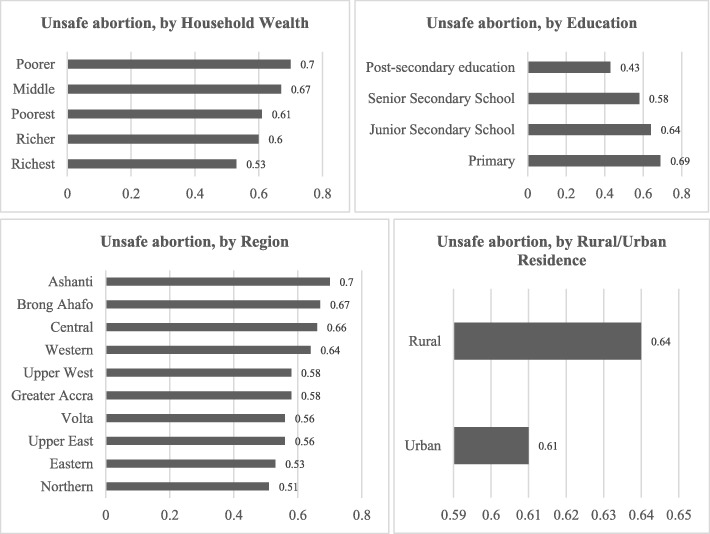
Bivariate Correlations between Unsafe Abortion and Selected Socioeconomic Factors

Bivariate correlations are examined between unsafe abortions in Ghana and selected women’s socioeconomic characteristics. These are presented in Fig. 2. Unsafe abortions are higher among women who belong to poorer households and who have little or no education. Examining rural-urban characteristics, it is observed that unsafe abortions are more common among women who reside in rural areas. Finally, examining regional differences, unsafe abortions are highest in the Ashanti region, followed by the Brong Ahafo region. These are regions with generally high abortion rates in the country. Regions with the lowest proportion of women who have undertaken unsafe abortions include the Northern region, followed by the Eastern region.

### Multivariate analyses

Table 2 presents the results of the multivariate probit analyses of the likelihood of women obtaining an abortion in the last 5 years preceding the GMHS; the probability of obtaining a safe abortion; and the likelihood of higher self-efficacy in abortion decision-making among women in Ghana. It is important to state that it is difficult to make causal claims about the nature of the relationships between women’s abortion experience, self-efficacy status and model explanatory variables. Descriptive analyses of these relationships have therefore been provided in the preceding section, in addition to a series of robust correlations in this section through the probit model specification. This is aimed at facilitating the examination of the nature of the existing relationships.

**Table 2:** Marginal Effects of Abortion, Safe Abortion and Self Efficacy on Explanatory Variables, GMHS, 2017

	Abortion in last 5 years	Safe Abortion	Abortion Self-Efficacy
Woman’s age	0.00	0.03	0.00
	(−1.00)	(1.08)	(0.3)
Age squared	0.00	0.00	0.00
	(0.46)	(−0.65)	(−0.43)
Partner is present	−0.02**	− 0.05	− 0.04***
	(−2.23)	(−1.23)	(−3.17)
Household size	− 0.00**	0.03***	0.00
	(−2.18)	(3.50)	(0.02)
*Education (None/primary is base)*			
Junior secondary school	−0.01	− 0.03	0.02*
	(−0.89)	(−0.48)	(1.67)
Senior secondary education	−0.01	0.00	0.02
	(−1.00)	(0.00)	(1.08)
Post-secondary education	−0.04**	0.17**	0.00
	(−2.38)	(1.98)	(0.17)
Children ever born	−0.02***	− 0.02	0.00
	(−5.91)	(−0.93)	(−0.56)
Polygyny	0.04***	0.08	0.03*
	(2.95)	(1.25)	(1.74)
*Religion (Catholic is base)*			
Muslim	−0.04**	0.07	− 0.04**
	(−2.23)	(0.80)	(−2.23)
Other Christian	−0.01	−0.05	0.00
	(−0.42)	(−0.73)	(0.2)
Traditionalist/ Athiest	−0.01	−0.16	− 0.02
	(−0.43)	(− 0.88)	(− 0.51)
*Wealth Quintile (base is Poorest)*			
poorer	0.06***	0.00	0.00
	(3.42)	(0.00)	(0.12)
middle	0.08***	0.00	0.01
	(4.40)	(−0.03)	(0.49)
richer	0.09***	0.03	0.02
	(4.60)	(0.26)	(0.66)
richest	0.09***	0.02	0.01
	(3.94)	(0.14)	(0.30)
Bank account	0.00	0.10**	0.04***
	(−0.06)	(2.34)	(3.01)
Ownership of radio	0.00	−0.02	0.00
	(−0.40)	(−0.46)	(0.20)
Ownership of TV	0.01	−0.03	0.03*
	(0.58)	(−0.39)	(1.65)
urban	0.00	0.02	−0.02
	(−0.12)	(0.39)	(−1.43)
Access to health facility	−0.01	0.02	0.04***
	(−0.60)	(0.42)	(3.25)
Perceptions_Abortion legality	0.00	0.13**	0.07***
	(0.25)	(2.12)	(4.74)
NHIS (covered)	−0.02**	0.09**	–
	(−2.11)	(2.42)	
*Ethnic Groups (Mole-Dagbani is base)*			
Akan	0.02	0.08	0.00
	(1.17)	(0.93)	(−0.22)
Ga	0.01	−0.06	0.00
	(0.58)	(−0.49)	(0.13)
Ewe	0.00	0.01	−0.02
	(0.22)	(0.08)	(−0.66)
Other northerner	0.04**	−0.08	−0.05***
	(2.22)	(−0.91)	(−2.78)
*Regions (Northern is base*)			
Western	0.14***	−0.13	−0.01
	(5.83)	(−0.81)	(−0.47)
Central	0.11***	−0.21	0.14***
	(4.26)	(−1.31)	(4.91)
Greater Accra	0.15***	−0.13	0.05
	(5.97)	(−0.79)	(1.54)
Volta	0.13***	−0.02	−0.05
	(4.55)	(−0.13)	(−1.47)
Eastern	0.11***	−0.08	−0.14***
	(4.51)	(−0.48)	(−4.75)
Ashanti	0.13***	−0.24	0.13***
	(5.49)	(−1.58)	(5.19)
Brong Ahafo	0.13***	−0.17	0.11***
	(5.25)	(−1.11)	(4.17)
Upper East	0.04*	−0.36**	−0.05**
	(1.68)	(−2.03)	(−2.17)
Upper West	0.07***	−0.09	0.12***
	(2.82)	(−0.54)	(5.25)
# of Observations	6099	609	6735

T-statistics in parentheses: *- *p* < 0.10; **- *p* < 0.05; ***-*p* < 0.01

The presence of a male husband or partner in the household has important implications for women’s self-efficacy and their experience of abortion. These findings are highly significant at the 1% level. Women who live with their partners are less likely to have abortions and also less likely to feel that they can take sole decisions relating to abortion. This effect of partner presence is consistent with existing literature. Research indicates that a woman’s decision to abort, often stems from a lack of support from partners [35, 46]. Women who live away from their partners also appear to feel more capable of taking sole decisions on abortion, which is likely to contribute to actual experiences of abortion. It is important to recognize that given the noted higher desired fertility among men, compared to women, in developing countries [21], abortion may cause challenges and stress within the relationship, leading to separation or divorce [8, 29, 41]. This indicates some potential for reverse causality in the relationship between partner presence and abortion.

There appears to be a negative correlation between household size and abortion, and a positive and highly significant (at 1%) relationship between household size and safe abortions. This finding may be related to network effects that may influence a woman’s abortion-seeking behavior and practices [13, 44]. Women who reside in polygynous households are more likely to have higher abortion self-efficacy, although this relationship is only weakly significant at the 10% level; these group of women are also significantly more likely to have abortions. Women who reside in polygynous households may be less easily monitored by their husbands, especially in cases where they reside in different compounds, a situation which is likely to increase their feelings of independence in reproduction decision-making and the likelihood of having (safe) abortions.

Women’s education achievement also appears to be important [17]. Compared to women with only primary education, women with post-secondary education are significantly less likely to have abortions but are more likely to opt for safe abortions if they do get abortions [49]. This may be explained by the greater access to knowledge and financial resources which may allow them to access better care [49]. Women with junior secondary school education are also more likely to possess abortion self-efficacy, compared to women with none or only primary education, although these results are only weakly significant at the 10% level.

Higher parity women are significantly less likely to have abortions [49]. This may be because these women often have less educated partners with higher desired family size. Where male partners wield a lot of control over reproductive decision-making, the likelihood of abortion may be low [19, 20]. With respect to religion, Muslim women are less likely to have abortion self-efficacy and also less likely to have abortions, compared to Christians. This may be explained by the lower desired fertility among Christians and therefore the higher proclivity for abortion [2, 11]. The lower self-efficacy noted among Muslim women may also explain their lower likelihood of abortion.

The effects of financial resources and socioeconomic status are interesting- although increasing household wealth is highly significant and associated with increasing likelihoods of a woman having had an abortion [49], it is a woman’s personal ownership of a bank account that affects significantly her decision concerning a safe/unsafe abortion method. The ownership of a personal bank account is also a more critical determinant of abortion self-efficacy, compared to household wealth. Therefore, women who have bank accounts and a larger sense of financial freedom are more likely to have higher self-efficacy and are more likely to opt for safer abortion methods.

Mass media is an important determinant of abortion self-efficacy and women who own television sets are more likely to have higher abortion self-efficacy. This is because women may be better exposed to western ideas and messages that encourage independence and greater autonomy. Access to health facilities is also important and women are more likely to have better self-efficacy if there are limited challenges to accessing health care. This finding is highly significant at the 1% level. The study also finds that women’s perceptions about the laws surrounding abortion practices in Ghana is an important factor. Women who perceive that abortion is legal are significantly more likely to have better self-efficacy. They are also more likely to opt for safe methods of abortion [49]. Access to health financing is also important- women who report that they are covered by the county’s national health insurance scheme are less likely to have had an abortion, but in the event that they do, they tend to have had safer abortion procedures [23]. With respect to regional differences, compared to women in the Northern region, women who reside in other regions are more likely to have had an abortion in the 5 years preceding the survey. Of the women who have had abortions, women in the Upper East regions are less likely to have employed a safe abortion method. Compared to women in the Northern region, women in the East and Upper East region are less likely to have abortion self-efficacy. Women in the Central, Ashanti, Brong Ahafo and Upper West regions are however more likely to have higher abortion self-efficacy.

As illustrated in Table 3, it is important to note that in all cases, the group of women who have better self-efficacy are the same group of women likely to opt for safe abortion methods.

**Table 3:** Ghanaian Women Profiles for Abortion, Safe Abortions and Abortion Self-Efficacy

Profile	Abortions	Safe Abortions	Self-Efficacy
Partner presence	↓		↓
Polygyny	↑		↑
Christian	↑		↑
Large households	↓	↑	
Highly educated	↓	↑	↑
Wealthy	↑		
Personal bank account		↑	↑
NHIS coverage	↓	↑	
High fertility	↓		
Perceive abortion to be legal		↑	↑
Access to mass media			↑
Access to health facilities			↑

This may be indicative of a basic correlation, at least, between women’s self-efficacy and safe abortion experiences, therefore emphasizing the need to better understand the factors that underlie women’s self-efficacy. Paying attention to these factors could have important implications for women’s self-efficacy and by extension, their probabilities of opting for safe abortion practices in the future. This could further have impacts on the incidence of maternal morbidity and mortality in the country.

## Conclusion

Although abortion rates in Ghana have remained constant at 10% between 2007 and 2017, the proportion of women who are having unsafe abortions has increased from 45% in 2007 to 62% in 2017. In Ghana, more than 1-in-10 pregnancy deaths are as a result of unsafe abortion practices.

The most significant (at 1 or 5% level) predictors of safe abortions in Ghana appear to be the size of the household, post-secondary education, a woman’s ownership of a bank account, NHIS coverage and her perceptions about the legality of abortions in the country. At 1% significance levels, the most important predictors of abortion self-efficacy were the absence of a male partner, women’s ownership of a bank account, access to health facilities, women’s perception of the legality of Ghana’s abortion laws and non-northern ethnicity.

A number of policy applications may be derived from this research. Descriptive statistics indicate that unsafe abortions are common among women with lower education and wealth status, who may be easily overlooked in interventions aimed at reducing the incidence and consequences of unsafe abortion practices. Given the focus on universal health coverage, it is essential that all women have adequate access to necessary and safe reproductive health services. Maps of Ghana also indicate clustering of abortion in particular areas of the country, which may present opportunities for policy focus and interventions targeting.

Financial inclusivity through increased access to bank accounts should continue to remain high on the agenda, as it promotes a woman’s feelings of control over her own reproductive health. Financial independence also allows women to make safer choices regarding issues of their reproductive health. It may be important to pay more attention to counselling services and couple management in order to reduce the incidences of couple separation, family instability and abortions in the country. Although the laws in Ghana prohibit abortion, unless in special circumstances, women who perceive abortion to be legal appear to be more likely to opt for safe abortions. This is a finding which should be considered carefully. It appears that when women do not feel that they are breaking any laws, they are more likely to be more responsible in their abortion decisions, limiting the potential occurrence of maternal morbidity and mortality occurrences from unsafe abortion practices.

There are interesting findings of the effect of the national health insurance scheme (NHIS) on abortion experiences of women. Women who are covered by the scheme are less likely to have abortions. However, in cases where they do have abortions, they are more likely to use safe methods. This may be explained by the current structure of the NHIS. Although the NHIS does not cover abortions, it does cover health care services for complications arising out of abortion operations. It has been suggested recently by some reproductive health non-governmental organisations (NGOs) and civil service organizations that the current structure may put women at unnecessary risk for negative health consequences and increase the financial burden on the scheme. It is therefore proposed by these groups and others, that the NHIS simply cover abortion services (legal and illegal) for women in the country. From a reproductive health perspective, this may be a proposition worth considering- women who are covered by the NHIS are less likely to suffer negative consequences of unsafe abortions.

Other policy recommendations include the need to expand access to family planning services, particularly given the concern that women in Ghana may be using abortion services as a means of contraceptive. This is disturbing, given the potential negative health implications of abortions identified in the literature.

Abortion self-efficacy may have important implications for women’s abortion decision-making processes, particularly the choice of a safe abortion method. Factors such as women’s education, access to financial resources, mass media, health facilities and the abortion law have been established as important correlates of abortion self-efficacy. Policies to improve socioeconomic status of women and health access may therefore have important implications for women’s self-efficacy, use of safe abortion methods and by extension, their reproductive health outcomes.

## Data Availability

Original data used for this study is available upon request from the Demographic and Health Survey Program.

## Abbreviations

D&C: Dilation and Curettage
GIS: Geographic Information System
GMHS: Ghana Maternal Health Surveys
MVA: Manual vacuum aspiration
NGO: Non-governmental organisations
NHIS: National health insurance scheme
PCA: Principal component analysis
WHO: World Health Organisation
